# Anæsthesia: The Promise of Future Refinement

**Published:** 1919-05-31

**Authors:** 


### ANÆSTHESIA.

### The Promise of Future Refinement.

One of the clearest lessons derived from war experiences comes from realisation of the close relationship between methods of anæsthetic administration and the mortality in surgical cases. Diverse opinions are expressed as to the number of deaths at clearing stations, etc., in the earlier years of the fighting which could be considered as preventable had the present anæsthetic methods been in vogue at the time, but one hesitates to accept extreme views. There is no doubt, however, that great advances have been made, both in the selection of methods appropriate to particular types of case and in improvement in the technique of actual administration. Therefore, in the future surgery of this country, we must look for something more than the routine application of special types, either fashionable for the moment, or employed by men who favour them from personal choice or expertness. There must be full co-ordination between the clinical aspects of the case and the possible advantages or disadvantages of each means of anæsthesia. War surgery has not been confined to traumatic conditions alone; every type of work has been encountered, and it is therefore of great interest to trace the general tendency of these times as providing a guide to the future, not only in the theatres of our great hospitals, but in the most provincial operating work.

Generally speaking, chloroform has been discarded, although in mixtures, as warmed A.C.E. and C.E. vapours, etc., it is still employed by many surgeons in cases for which, by past experience, we know it to be suitable, or from the fact that details of newer methods take time to become general knowledge. For work upon chronic or sub-acute conditions, minor traumatic states, and a host of others in which such methods have long been in use, it is pointless to suggest that any great improvement can be expected if they are superseded, but it is with lesions, such as those near vital structures, accompanied by toxæmic states, shock, concurrent infection, gross injury, or requiring long and extensive operation, that we must take pains to select just that anæsthetic which will lessen mortality and reduce morbidity to the greatest extent.

### Gas and Oxygen.

Crile, Marshall, Gwathmey, and others have given us the results of considerable experience in this method. The sequence of gas and oxygen, with or without ether, is steadily gaining favour, and appears likely to become a favourite general method for all cases where local anæsthesia alone is impracticable. Also in combination with the regional method it has an immense field of utility where more perfect relaxation than gas and oxygen alone can give is required. In abdominal surgery, where a chest condition, such as "gassing," is incidental, it is found to be particularly useful, since both open ether and chloroform are here absolutely contra-indicated.

In the past, apparatus has, however, been both complicated and expensive. Apart from the liability of parts to get out of order, there were difficulties in supplying gas at uniform pressure, difficulties in having at hand a sufficient supply of gas—and, to some extent, these are with us still, particularly in adopting the method for other than institutional use. But recently several modifications have been devised—notably, one by the American Red Cross workers, which reduces the technical difficulties of administration even if the whole apparatus has not become more portable. Subsidiary ether supply is arranged to give the required percentage with a sight-feed control and, by this combination, it is claimed that any degree of relaxation can be obtained at the required period. Throughout, the patient is a relatively sure index, irrespective of apparatus, and if an even flow of gases with a dependable percentage composition be mechanically maintained the major need is supplied. To judge from published records, all who have extensively used this method are strong in its praise, not only for the period of actual operation, but on account of its lack of bad after-effects, and reduction of post-operative shock.

### Anæsthesia by Oral Administration.

As a mild form of anæsthesia this relatively new method has a number of advantages. Not only is the time of the anæsthetist saved, but this type of administration may be safely made after a meal and without the customary preparation of the patient. Capt. Gwathmey, of the U.S. Medical Service, finds that a preparation containing fifty per cent. ether in liquid petroleum administered by the mouth is a safe general analgesic, and is never accompanied by the stomach effects, nausea, and vomiting, which so frequently follow inhalation methods. He explains that the unpleasant taste may be readily obviated by intermittent sips of port wine. There is no need to remove the patient from his bed, and, supplemented by local or very light inhalation anæsthesia, or a morphia injection when necessary, the method can be made to embrace short surgical operations. A variety of formulæ has been used. One by Major Marshall at No. 17 C.C.S. was:—

| | |
|---|---|
| Ether ... ... ... ... ... | ounces 1½ |
| Chloroform... ... ... ... ... | minims xx |
| Liq. paraffin q.s. ad ... ... ... | ounces 4 |

given by mouth twenty minutes before operation. The patient lay back with a towel over his face, to induce a kind of rebreathing, and in 40 to 50 minutes was produced a satisfactory analgesia which could be reinforced at will by accessory methods. The idea seems to be of great promise for use in the innumerable cases met with in civil work where pain sufficient to distress must be caused, considerable time taken, and yet the use of a full anæsthetic impossible for one of the many reasons frequently encountered under such circumstances. Also in dressing severe compound fractures, extensive

**Anæsthesia**—(*continued*).

or multiple injuries and all instances where the minimum of movement is advisable, oral methods would be of immense utility.

SCOPOLAMINE-MORPHINE.

Considerable value has been claimed for scopolamine-morphine narcosis in the field as preparatory to emergency surgery, such as preliminary work on compound fractures, dislocations, ligature of vessels, etc. It is stated that if scopolamine gr. $\frac{1}{100}$ and morphia gr. $\frac{1}{6}$ are given hypodermically at once and repeated thirty minutes later, the patient reaches a satisfactory condition for any of this class of operation, in even the severest cases a small amount of inhaled anæsthetic at the appropriate moment being all that is required. Extension of the method can be made to include much major surgical work, upon exactly the same principle as the much-discussed "twilight sleep." Naturally, accessory ether or C.E. is frequently necessary, but those who have developed the technique under war conditions point out that there are many advantages over the simple preparation and inhalation measures. With or without adjuncts, scopolamine-morphine confers a surgical sleep which continues long after the operation is finished, and from which the patient wakes able readily to take liquid nourishment. Nausea, and vomiting, as stated, are largely inhibited in ninety-five per cent. of the cases, and when present are not remembered by the patient. So-called anoci association is conferred, and following the principles of the obstetric work some excellent results have undoubtedly been obtained.

SPINAL ANALGESIA.

For the intra-thecal methods in war surgery, no great novelty in technique can be claimed. The majority of those who have used it appear to favour a four per cent. solution of novocain and this has been extensively employed for painful dressings and wound closures below the waist line. Perhaps its greatest utility has been during the rush periods at C.C.S. or base, but as was pointed out during the discussions at the recent B.M.A. meeting, there is a distinct fall in blood pressure even when injection is made in patients not already suffering from shock. This was confirmed by Crile by experiments on animals and unless such effect can be prevented a definite objection appears. The psychic factor is also of importance under war as well as home conditions, although it may be largely overcome by preliminary morphia, nitrous oxide, or light ether anæsthesia, American writers are enthusiastic in praise of the spinal method, and this may be indirectly due to the improved medical organisation and opportunity prevailing in the later years of war, but in any case it may be said that this means of approach to the nervous system has proved its definite advantages in acute lung cases, conditions with renal infection and threatened uræmia, bad cardiac states, and many other instances. On the whole, however, war has not brought any striking developments in this direction.

LOCAL ANÆSTHESIA.

On the other hand, considerable extension of local and regional methods has resulted. Either alone or in combination with light general anæsthesia, procedures along the lines of progressive infiltration and nerve-blocking, which practically originated with the suggestions of Crile a few years ago, have been successfully performed in a great number of major operations. Many reports of completely satisfactory amputations at the thigh or leg, trephinings, transplantation of bone and cartilage, plastic operation, removal of foreign bodies, etc., have come to hand, and, although the details of new modifications and the *pros* and *cons* of their employment would occupy more space than is here available, one may say with confidence that we expect to see widespread application of these ideas in the near future. In the stress of war emergency they have been invaluable, and, in common with the various means already described, give promise of much future refinement in practice of the anæsthetist's art.

